# Editorial: Bronchopulmonary Dysplasia: Past, Current and Future Pathophysiologic Concepts and Their Contribution to Understanding Lung Disease

**DOI:** 10.3389/fmed.2022.922631

**Published:** 2022-07-07

**Authors:** Andrew Bush, Anne Hilgendorff

**Affiliations:** ^1^Imperial Centre for Paediatrics and Child Health, London, United Kingdom; ^2^National Heart and Lung Institute, London, United Kingdom; ^3^Royal Brompton and Harefield NHS Foundation Trust, London, United Kingdom; ^4^Center for Comprehensive Developmental Care (CDeC^*LMU*^) at the Interdisciplinary Social Pediatric Center, Department of Pediatrics, Dr. von Hauner Children's Hospital, University Hospital, LMU Munich, Ludwig-Maximilians University, Munich, Germany; ^5^Institute for Lung Health and Immunology and Comprehensive Pneumology Center, Helmholtz Zentrum München, Munich, Germany; ^6^German Center for Lung Research (DZL), Giessen, Germany

**Keywords:** neonate, preterm, chronic lung disease, bronchopulmonary dysplasia, long-term morbidity

Pulmonary disease arising from both pre- and immediate postnatal adverse events affecting the developing lung was first described around 50 years ago by Northway and colleagues (1). By today's standards, the disease termed “bronchopulmonary dysplasia” (BPD) was seen in relatively mature babies weighing over 2 kg at birth, who were ventilated with higher pressures and at slower rates than would be acceptable today. Much has happened since then.

Whereas, the initial or so called “old” BPD (Northway) was characterized by a pronounced effect on the airways, albeit with an element of failure of alveolar development, BPD today (“new” BPD) is characterized by lung hypoplasia driven by dysregulated growth factor signaling (2) in the context of extensive matrix remodeling and a pronounced inflammatory response (3, 4). This picture arises because neonatologists worldwide now salvaging babies weighing <500 g at birth.

Despite improved perinatal care, and most likely reflecting the changing picture of structural and functional consequences of lung injury in this patient cohort, the overall rates of chronic lung disease in the preterm infant have not decreased significantly and BPD remains the most common morbidity of prematurity (5–7). Adding another level of complexity, the validity and utility of commonly used BPD definitions have been questioned and most prediction models for BPD only hold limited value for clinical use (8–10). Likewise, contemporary changes in the perinatal management of infants, such as the use of high-flow nasal cannula and less aggressive neonatal resuscitation, limit the application of prior definitions, and may result in further misclassification of the disease (11).

Unresolved as of today is the presumptive presence of different disease endotypes with variable contributions of airway pathology, matrix remodeling and pulmonary vascular disease, all covered by the umbrella term BPD.

As clinical understanding of the ever-changing picture of BPD is limited, conceptual insights derived from careful clinical observations and validated scientific findings in human babies and preclinical animal models were and are needed to improve clinical standards. As a consequence, the use of antenatal steroids and postnatal surfactant have become routine and resuscitation has become gentler, especially with the previous imperative to get the baby fully oxygenated immediately after birth having been removed. The use of supplemental oxygen to maintain oxygen saturation in an evidence based low-normal range (12)—depending on lung vascular and extrapulmonary complications—is now known to be important as is the avoidance of unnecessarily high oxygen concentrations even for short periods of time. In addition to the reduction or prevention of oxygen toxicity, ventilation regimens have been profoundly revised with lower ventilation pressures and higher ventilation rates than were applied in the early years of neonatology (13, 14).

As important for minimizing the risk of sustained lung injury is the controlling of postnatal infections and the optimization of fluid balance (15, 16) and nutrition while preventing fluid overload and normalizing somatic growth. Prenatal risk factors including intra-amniotic infections and growth retardation are known to contribute to disease development in the structurally and functionally immature lung, but are—despite improved treatment regimen to initially support lung function and potentially maturation (17, 18)—still poorly controlled as of today and long-term benefits of existing therapies remain unclear.

Identification of the risk factors outlined above has helped to inform concepts of pre- and postnatal management although many challenges are only partially understood and therefore still unmet. Inevitable risks for BPD development remain including the higher risk for males. As both experimental and clinical studies revealed, other genetically determined risk factors are important, and an initial report has highlighted the involvement of a multitude of genes implicated in BPD (19).

Similarly challenging, new therapeutic developments that have been and will be highly beneficial for babies and their families, but are—as is well-known—accompanied by iatrogenic complications and change the pattern of disease in survivors. Two examples are the use of systemic corticosteroids in pre- and postnatal treatment regimens (17, 20, 21) and the careful consideration of potentially longer periods of oxygen exposure (22) as a tradeoff for a reduced period of invasive ventilation.

Nonetheless, even if neonatal practice was perfect, being born preterm is enough to lead to long term respiratory consequences, independent of any iatrogenic complications (23, 24).

In parallel with the change in the nature of BPD, tools for investigation and monitoring have become increasingly sophisticated. These include infant and pre-school lung function, including sensitive tests such as multi-breath washout, and imaging, initially with high-resolution computed tomography, and now increasingly with magnetic resonance imaging. Next to the need for further refinement of structural assessment using advanced imaging technologies while avoiding radiation exposure or general anesthesia, unmet challenges include the lack of biomarkers allowing for early disease detection and subsequent monitoring of progression. Here, multiomic technologies and systems biology may be of help including further sophisticated probing of potential BPD endotypes, that could allow individualized and maybe pathway specific treatment and monitoring approaches.

Adding to the complex disease picture, knowledge about co-morbidities is sparse but will—with increasing long-term survival—likely gain importance. The understanding of the late complications of BPD will inform neonatal practice, by analogy with cystic fibrosis, where long survival has led to the realization of the importance of detecting diabetes and bone disease in children and instituting preventive and treatment strategies. We have found out much about cardiovascular and metabolic morbidity and mortality in survivors; low first second forced expired volume (FEV_1_) is a marker of increased risk in the normal term (25, 26), but also in BPD survivors (27, 28). Is our monitoring for diabetes and hyperlipidaemia adequate? These and other questions show that BPD is an illustration of the importance of not living in “developmental silos,” but taking a whole life course view of traditional “pediatric” diseases.

In light of these considerations, a further challenge is engaging adult thoracic physicians in following these children up to determine the long-term consequences of BPD. Unfortunately, and in contrast to the tremendous investment in intensive care and the immediate aftermath, by and large respiratory follow up has not happened, despite the known respiratory morbidity in survivors (29). The survivors are thus often likely to be given inappropriate therapy such as treatment with inhaled corticosteroids for “asthma-like” phenotypes in preterm infants, despite the compelling evidence of a non-inflammatory airway phenotype in most BPD survivors with wheeze. Many will not attain normal spirometric values and will thus be at risk for being diagnosed with chronic obstructive pulmonary disease (COPD) (30). In addition, adult patient care often neglects early life events (31), increasing the risk for survivors of prematurity to be falsely combined under the COPD umbrella together with chronic smokers, although they may have a very different endotype leading to the end stage of a reduced FEV_1_/forced vital capacity (FEV_1_/FVC) ratio. This knowledge—together with the exchange of insights into potentially common pathways in neonatal and adult lung disease and the contribution to regeneration strategies informed by specialists in lung development—should lead to a fruitful exchange between experts and care givers. But as the face of the disease is changing, so a new generation of problems is on the way.

In face of all these achievements and remaining challenges, it becomes increasingly clear, that BPD is a dynamic disease that will be changing with neonatal practice and it would be unwise to assume that there will not be further important developments in the years ahead.

Understanding BPD therefore requires novel concepts stemming from clinically relevant experimental and translational approaches that undergo holistic, multi-layered evaluation to determine their utility for disease understanding and clinical care (see Figure 1). Showcasing the complexity of events is the need to understand cellular crosstalk in the developing alveolar niche while considering the impact of the surrounding scaffold to gain insight into repair and regeneration capacities as well as mechanisms of progression to chronic disease. Premature ageing and the implications of senescence are poorly understood but likely play an important role in determining the outcomes. Questions that concern e.g., the mechanisms behind a “switch off” of a specific cellular developmental capacity in lung development and their later “switch on” in adolescence or adulthood have yet to be explored.

**Figure 1:**
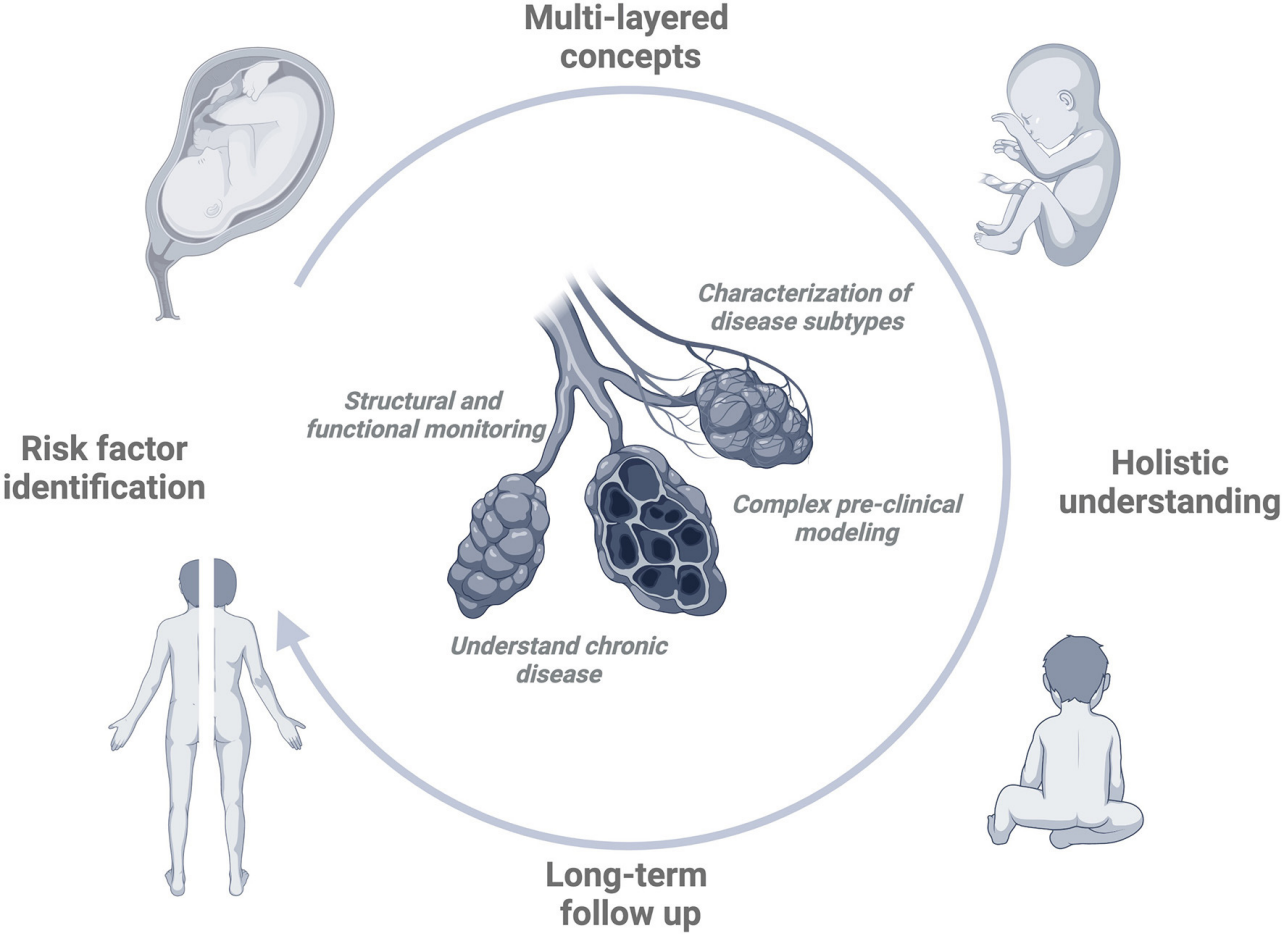
BPD circle. In order to develop effective and comprehensive strategies to treat or prevent BPD, the identification and understanding of important pre- and postnatal contributors, i.e., risk factors is key. In order to generate a more holistic understanding of the disease, the development of multilayered pathophysiological concepts involving different areas of expertise will help us to move beyond over-simplified models of cause and consequence. In order to validate the relevance of proposed effects, insights obtained during long-term follow-up need to translate into knowledge about initial injury and risk factor impact. Bearing these goals in mind, the improvement of our understanding of the disease is supported by the development of diagnostic tools to better characterize lung structural changes and their functional consequences. The identification of (potential) disease ‘subtypes' may allow us to understand more differentiated relationships between risk factor impact and clinical outcome. Complex, pre-clinical modeling is a prerequisite to drawing clinically relevant conclusions while considering the processes that mark the ‘tipping point' of resolution to progression to chronic disease. Created with BioRender.com.

In order to inspire the development of novel, broader concepts, a comprehensive overview over the state of knowledge in the BPD field is needed covering ongoing research and clinical developments.

We have been privileged to work with a stellar group of authors and commentators to put this “BPD Research Topic” together. The content includes a comprehensive overview about available (and feasible) animal models to unravel disease mechanisms and perform preclinical studies (32, 33), insight into critical cell populations, the lung matrix and their complex interaction as determinators of the pulmonary landscape (2), and models of the nature and causation of lung injury reviewing the most critical pathophysiologic mechanisms (34, 35). Other manuscripts reflect on the role of new diagnostic and treatment concepts (36–39).

By combining specialist chapters with overview commentaries—often from authors who are experts in other fields of lung disease—we have furthermore aimed at giving a broader context to BPD with an “outside” view complementing the perspective from “within the NICU” (24).

We believe that the Research Topic on bronchopulmonary dysplasia provides a rounded and comprehensive picture of a disease that significantly determines quality of survival throughout the life course in one of the largest pediatric patient groups. It should paradigm for understating long-term consequences of early (multiple hit) injury.

## Author Contributions

AB and AH contributed as editors to the Research Topic on bronchopulmonary dysplasia by conceptualizing and supervising the content, inviting the contributing authors, and by writing the Editorial. Both authors contributed to the article and approved the submitted version.

## Conflict of Interest

The authors declare that the research was conducted in the absence of any commercial or financial relationships that could be construed as a potential conflict of interest.

## Publisher's Note

All claims expressed in this article are solely those of the authors and do not necessarily represent those of their affiliated organizations, or those of the publisher, the editors and the reviewers. Any product that may be evaluated in this article, or claim that may be made by its manufacturer, is not guaranteed or endorsed by the publisher.

## References

[1] Northway WH Rosan RC Porter DY. Pulmonary disease following respirator therapy of hyaline-membrane disease. Bronchopulmonary dysplasia. N Engl J Med. (1967) 276:357–68. 10.1056/NEJM196702162760701 5334613

[2] Oak P Hilgendorff A. The BPD trio? Interaction of dysregulated PDGF, VEGF, and TGF signaling in neonatal chronic lung disease. Mol Cell Pediatr. (2017) 4:11. 10.1186/s40348-017-0076-8 29116547 PMC5676585

[3] Shahzad T Radajewski S Chao CM Bellusci S Ehrhardt H. Pathogenesis of bronchopulmonary dysplasia: when inflammation meets organ development. Mol Cell Pediatr. (2016) 3:23. 10.1186/s40348-016-0051-9 27357257 PMC4927524

[4] Mizikova I Morty RE. The extracellular matrix in bronchopulmonary dysplasia: target and source. Front Med. (2015) 2:91. 10.3389/fmed.2015.00091 26779482 PMC4688343

[5] Stoll BJ Hansen NI Bell EF Shankaran S Laptook AR Walsh MC. Neonatal outcomes of extremely preterm infants from the NICHD Neonatal Research Network. Pediatrics. (2010) 126:443–56. 10.1542/peds.2009-2959 20732945 PMC2982806

[6] Shah PS Sankaran K Aziz K Allen AC Seshia M Ohlsson A. Outcomes of preterm infants <29 weeks gestation over 10-year period in Canada: a cause for concern? J Perinatol. (2012) 32:132–8. 10.1038/jp.2011.68 21593814

[7] Stroustrup A Trasande L. Epidemiological characteristics and resource use in neonates with bronchopulmonary dysplasia: 1993-2006. Pediatrics. (2010) 126:291–7. 10.1542/peds.2009-3456 20643728

[8] Shennan AT Dunn MS Ohlsson A Lennox K Hoskins EM. Abnormal pulmonary outcomes in premature infants: prediction from oxygen requirement in the neonatal period. Pediatrics. (1988) 82:527–32. 10.1542/peds.82.4.527 3174313

[9] Jobe AH Ikegami M. Prevention of bronchopulmonary dysplasia. Curr Opin Pediatr. (2001) 13:124–9. 10.1097/00008480-200104000-00006 11317052

[10] Walsh M Laptook A Kazzi SN Engle WA Yao Q Rasmussen M. A cluster-randomized trial of benchmarking and multimodal quality improvement to improve rates of survival free of bronchopulmonary dysplasia for infants with birth weights of less than 1250 grams. Pediatrics. (2007) 119:876–90. 10.1542/peds.2006-2656 17473087

[11] Poindexter BB Feng R Schmidt B Aschner JL Ballard RA Hamvas A. Comparisons and limitations of current definitions of bronchopulmonary dysplasia for the prematurity and respiratory outcomes program. Ann Am Thorac Soc. (2015) 12:1822–30. 10.1513/AnnalsATS.201504-218OC 26397992 PMC4722827

[12] Lui K Jones LJ Foster JP Davis PG Ching SK Oei JL. Lower versus higher oxygen concentrations titrated to target oxygen saturations during resuscitation of preterm infants at birth. Cochrane Database Syst Rev. (2018) 5:CD010239. 10.1002/14651858.CD010239.pub2 29726010 PMC6494481

[13] Cools F Offringa M Askie LM. Elective high frequency oscillatory ventilation versus conventional ventilation for acute pulmonary dysfunction in preterm infants. Cochrane Database Syst Rev. (2015) 2015:CD000104. 10.1002/14651858.CD000104.pub4 25785789 PMC10711725

[14] Klingenberg C Wheeler KI McCallion N Morley CJ Davis PG. Volume-targeted versus pressure-limited ventilation in neonates. Cochrane Database Syst Rev. (2017) 10:CD003666. 10.1002/14651858.CD003666.pub4 29039883 PMC6485452

[15] Beeton ML Maxwell NC Davies PL Nuttall D McGreal E Chakraborty M. Role of pulmonary infection in the development of chronic lung disease of prematurity. Eur Respir J. (2011) 37:1424–30. 10.1183/09031936.00037810 20884745

[16] Oh W. Fluid and electrolyte management of very low birth weight infants. Pediatr Neonatol. (2012) 53:329–33. 10.1016/j.pedneo.2012.08.010 23276435

[17] Bassler D Shinwell ES Hallman M Jarreau PH Plavka R Carnielli V. Long-term effects of inhaled budesonide for bronchopulmonary dysplasia. N Engl J Med. (2018) 378:148–57. 10.1056/NEJMoa1708831 29320647

[18] McGoldrick E Stewart F Parker R Dalziel SR. Antenatal corticosteroids for accelerating fetal lung maturation for women at risk of preterm birth. Cochrane Database Syst Rev. (2017) 12:Cd004454. 10.1002/14651858.CD004454.pub4 33368142 PMC8094626

[19] Li J Yu KH Oehlert J Jeliffe-Pawlowski LL Gould JB Stevenson DK. Exome sequencing of neonatal blood spots and the identification of genes implicated in bronchopulmonary dysplasia. Am J Respir Crit Care Med. (2015) 192:589–96. 10.1164/rccm.201501-0168OC 26030808 PMC4595691

[20] Jefferies AL. Postnatal corticosteroids to treat or prevent chronic lung disease in preterm infants. Pediatrics. (2012) 109:330–8. 10.1542/peds.109.2.330 11826218

[21] Lemyre B Dunn M Thebaud B. Postnatal corticosteroids to prevent or treat bronchopulmonary dysplasia in preterm infants. Paediatr Child Health. (2020) 25:322–31. 10.1093/pch/pxaa073 32765169 PMC7395322

[22] Askie LM Darlow BA Finer N Schmidt B Stenson B Tarnow-Mordi W. Association between oxygen saturation targeting and death or disability in extremely preterm infants in the neonatal oxygenation prospective meta-analysis collaboration. J Am Med Assoc. (2018) 319:2190–201. 10.1001/jama.2018.5725 29872859 PMC6583054

[23] McGeachie MJ Yates KP Zhou X Guo F Sternberg AL Van Natta ML. Patterns of growth and decline in lung function in persistent childhood asthma. N Engl J Med. (2016) 374:1842–52. 10.1056/NEJMoa1513737 27168434 PMC5032024

[24] Singer D Thiede LP Perez A. Adults born preterm long-term health risks of former very low birth weight infants. Dtsch Arztebl Int. (2021) 118:521. 10.3238/arztebl.m2021.0164 33734986 PMC8503949

[25] Agusti A Noell G Brugada J Faner R. Lung function in early adulthood and health in later life: a transgenerational cohort analysis. Lancet Respir Med. (2017) 5:935–45. 10.1016/S2213-2600(17)30434-4 29150410

[26] Martinez FJ Foster G Curtis JL Criner G Weinmann G Fishman A. Predictors of mortality in patients with emphysema and severe airflow obstruction. Am J Respir Crit Care Med. (2006) 173:1326–34. 10.1164/rccm.200510-1677OC 16543549 PMC2662972

[27] Crump C Howell EA Stroustrup A McLaughlin MA Sundquist J Sundquist K. Association of preterm birth with risk of ischemic heart disease in adulthood. J Am Med Assoc Pediatr. (2019) 173:736–43. 10.1001/jamapediatrics.2019.1327 31157896 PMC6547251

[28] Crump C Sundquist J Winkleby MA Sundquist K. Gestational age at birth and mortality from infancy into mid-adulthood: a national cohort study. Lancet Child Adolesc Health. (2019) 3:408–17. 10.1016/S2352-4642(19)30108-7 30956154 PMC6691360

[29] Bolton CE Bush A Hurst JR Kotecha S McGarvey L. Lung consequences in adults born prematurely. Thorax. (2015) 70:574–80. 10.1136/thoraxjnl-2014-206590 25825005

[30] Vestbo J Edwards LD Scanlon PD Yates JC Agusti A Bakke P. Changes in forced expiratory volume in 1 second over time in COPD. N Engl J Med. (2011) 365:1184–92. 10.1056/NEJMoa1105482 21991892

[31] Bolton CE Bush A Hurst JR Kotecha S McGarvey L Stocks J. Are early life factors considered when managing respiratory disease? A British Thoracic Society survey of current practice. Thorax. (2012) 67:1110. 10.1136/thoraxjnl-2012-202637 22993167

[32] Chen S Rong M Platteau A Hehre D Smith H Ruiz P. CTGF disrupts alveolarization and induces pulmonary hypertension in neonatal mice: implication in the pathogenesis of severe bronchopulmonary dysplasia. Am J Physiol Lung Cell Mol Physiol. (2011) 300:L330–40. 10.1152/ajplung.00270.2010 21239535 PMC3064286

[33] Fujita M Mason RJ Cool C Shannon JM Hara N Fagan KA. Pulmonary hypertension in TNF-alpha-overexpressing mice is associated with decreased VEGF gene expression. J Appl Physiol. (2002) 93:2162–70. 10.1152/japplphysiol.00083.2002 12391106

[34] Thébaud B Goss KN Laughon M Whitsett JA Abman SH Steinhorn RH. Bronchopulmonary dysplasia. Nat Rev Dis Primers. (2019) 5:78. 10.1038/s41572-019-0127-7 31727986 PMC6986462

[35] Kalikkot Thekkeveedu R Guaman MC Shivanna B. Bronchopulmonary dysplasia: a review of pathogenesis and pathophysiology. Respir Med. (2017) 132:170–7. 10.1016/j.rmed.2017.10.014 29229093 PMC5729938

[36] Hafner F Kindt A Forster K Von Torne C Hauck SM Schubert B. Early risk stratification in preterm infants with Bronchopulmonary Dysplasia *via* pulmonary arterial flow measurements in MRI. Eur Respirat J. (2020) 56:4791. 10.1183/13993003.congress-2020.4791

[37] Förster K Ertl-Wagner B Ehrhardt H Busen H Sass S Pomschar A. Altered relaxation times in MRI indicate bronchopulmonary dysplasia. Thorax. (2020) 75:184–7. 10.1136/thoraxjnl-2018-212384 31048507

[38] Mairhörmann B Castelblanco A Häfner F Pfahler V Haist L Waibel D. Deep learning-based magnetic resonance imaging lung segmentation and volumetric marker extraction in preterm infants. medRxiv. (2021). 10.1101/2021.08.06.21261648

[39] Stoecklein S Hilgendorff A Li M Förster K Flemmer AW Galiè F. Variable functional connectivity architecture of the preterm human brain: impact of developmental cortical expansion and maturation. Proc Natl Acad Sci USA. (2020) 117:1201–6. 10.1073/pnas.1907892117 31888985 PMC6969522

